# Ternary MOF-Based Redox Active Sites Enabled 3D-on-2D Nanoarchitectured Battery-Type Electrodes for High-Energy-Density Supercapatteries

**DOI:** 10.1007/s40820-020-00528-9

**Published:** 2020-11-02

**Authors:** Goli Nagaraju, S. Chandra Sekhar, Bhimanaboina Ramulu, Sk. Khaja Hussain, D. Narsimulu, Jae Su Yu

**Affiliations:** 1 Institute for Wearable Convergence Electronics, Department of Electronic Engineering, Kyung Hee University, 1732 Deogyeong-daero, Gihung-gu, Yongin-si, Gyeonggi-do 17104 Republic of Korea; 2 Department of Chemical Engineering, College of Engineering, Kyung Hee University, 1732 Deogyeong-daero, Gihung-gu, Yongin-si, Gyeonggi-do 17104 Republic of Korea

**Keywords:** Metal–organic frameworks, Dual layers, Redox chemistry, Supercapattery, Renewable energy

## Abstract

Designing rationally combined metal–organic frameworks (MOFs) with multifunctional nanogeometries is of significant research interest to enable the electrochemical properties in advanced energy storage devices. Herein, we explored a new class of binder-free dual-layered Ni–Co–Mn-based MOFs (NCM-based MOFs) with three-dimensional (3D)-on-2D nanoarchitectures through a polarity-induced solution-phase method for high-performance supercapatteries. The hierarchical NCM-based MOFs having grown on nickel foam exhibit a battery-type charge storage mechanism with superior areal capacity (1311.4 μAh cm^−2^ at 5 mA cm^−2^), good rate capability (61.8%; 811.67 μAh cm^−2^ at 50 mA cm^−2^), and an excellent cycling durability. The superior charge storage properties are ascribed to the synergistic features, higher accessible active sites of dual-layered nanogeometries, and exalted redox chemistry of multi metallic guest species, respectively. The bilayered NCM-based MOFs are further employed as a battery-type electrode for the fabrication of supercapattery paradigm with biomass-derived nitrogen/oxygen doped porous carbon as a negative electrode, which demonstrates excellent capacity of 1.6 mAh cm^−2^ along with high energy and power densities of 1.21 mWh cm^−2^ and 32.49 mW cm^−2^, respectively. Following, the MOF-based supercapattery was further assembled with a renewable solar power harvester to use as a self-charging station for various portable electronic applications.

**Electronic supplementary material:**

The online version of this article (10.1007/s40820-020-00528-9) contains supplementary material, which is available to authorized users.

## Highlights

- Redox chemistry enabled Ni–Co–Mn (NCM)-based MOF nanoarchitectures are used as battery-type electrodes.
- The NCM-based MOFs demonstrate high areal capacity and good cycling stability.
- The fabricated hybrid supercapattery showed high energy and power densities of 1.21 mWh cm^−2^ and 32.49 mW cm^−2^, respectively.

**Electronic supplementary material:**

The online version of this article (10.1007/s40820-020-00528-9) contains supplementary material, which is available to authorized users.

## Graphical abstract

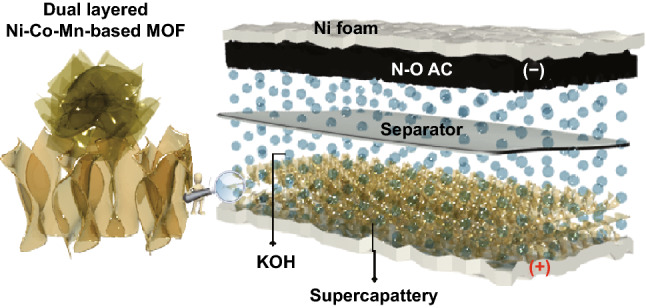

## Introduction

With the rapid growth of environmental issues, deteriorative fossil fuels and increasing energy demands, finding efficient methods to harvest intermittent natured renewable energy has greatly accelerated the development of perpetual and high-performance energy storage/conversion systems [1–5]. Among the various energy storage technologies, supercapacitors (SCs) have attracted tremendous attention by virtue of their fast charging-discharging capability, high power rate, and negligible safety risks [6–8]. The beneficial features of SCs have enabled distinctive applications in portable electronic and hybrid electric vehicles [9–11]. However, wide applications of SCs are still required to be magnified in terms of energy density, which is due to the limited potential window and capacitive performance of SCs [12–14]. One of the predominant approaches to escalate the energy density of device is to combine versatile charge storage mechanisms of capacitive-type (activated carbon) and battery-type (such as metal oxides) electrode materials in a curious hybrid paradigm, i.e., supercapatteries [15–17]. Utilizing the synergistic properties of these electrodes, the supercapattery system could exhibit high operating potential and capacity properties, thus leading to the superior energy density without losing their promising features of rate capability, cycling durability, and power density, respectively [18, 19]. However, the electrode materials with intrinsic synergistic properties should be selected to assist the enhancement of energy storage performance in supercapatteries [20, 21].

Recently, metal–organic frameworks (MOFs) have gained remarkable research interest in various technologies, owing to their high surface area, large pore volume, and tunability of their structure using numerous inorganic metal centers and organic linkers [22–24]. Moreover, the rich redox centers of these materials have another advantage to be directly used as an electrode material in energy storage systems, which paves an interesting attention toward superior charge storage [25]. Particularly, Ni- and Co-based MOF nanostructures involving organic–inorganic species have shown excellent capacity due to their higher electrochemical conductivity, richer redox reactions, and superior electrolyte accessible area, which enables high amount of active sites and high charge transfer capability [25, 26]. Compared to the solitary MOFs, the mixed MOF nanostructures have attracted great attention to further boost the energy storage performance in supercapatteries. The superior energy storage performance of the mixed MOF nanostructures is attributed to the change in environment of local coordination and electronic structure due to the doping of ternary metal cations [27, 28]. Moreover, the mixed metal species in MOFs increase the conductivity and redox chemistry rate resulted by the multiple oxidation states and they can act as an electron mediator to accelerate charge transfer via organic linker-to-metals to greatly exalt the energy storage performance [28–30]. On the other hand, the morphology of MOFs also played a crucial role in elevating the device performance. In particular, the MOF-based materials with hierarchical nanostructures could provide a credible solution to accelerate the capacity and rate performance owing to the enriched redox active sites, rapid electrolyte diffusion channels, and multi-electron transportation paths [26]. The various mixed MOFs with one-dimensional (1D), 2D, and 3D nanostructures have been reported, such as Co–Ni-MOFs@rGO hybrids, Ni–Co-MOF nanosheets, flower-like metallic MOFs (Co–Ni-MOFs), and porous Co-Mn-based MOF nanostructures, synthesized using various growth methods, which showed better energy storage properties and cycling stability [29, 31–33]. The time-consuming/high-temperature synthesis, limited electroactive sites, and rather poor redox chemistry of these solitary nanostructures have initiated researchers to develop rational combination of hierarchical redox-type MOFs with controlled geometries to enhance the energy density of supercapatteries.

In this work, we report a binder-free ternary Ni–Co–Mn-based MOF (NCM-based MOF) with hierarchical/dual-layered structures on nickel (Ni) foam using a polarity-induced solution-phase method. With the controlled growth time, the dual-layered NCM-based MOFs with 2D nanosheets as a bottom layer and randomly distributed 3D nanoflowers as a top layer were facilely integrated on Ni foam with robust adhesion. The synergistic features of the dual-layered NCM-based MOFs demonstrated superior electrochemical properties due to the increased electroactive sites, rapid electrolyte diffusion paths, and rich redox reactions in aqueous alkaline electrolyte (potassium hydroxide (KOH)). Moreover, the novel supercapattery system has been fabricated with dual-layered NCM-based MOFs as a battery-type and nitrogen–oxygen rich activated carbon (N–O AC) as a negative electrode with a piece of filter paper as a separator and KOH as an electrolyte in pouch-type configuration. The energy storage performance including energy and power densities of the assembled supercapattery was investigated. Additionally, a renewable solar energy has been effectively stored into the supercapattery system for various self-powered electronic applications.

## Results and Discussion

### Fabrication and Formation of Dual-Layered NCM-based MOFs

**Fig. 1:**
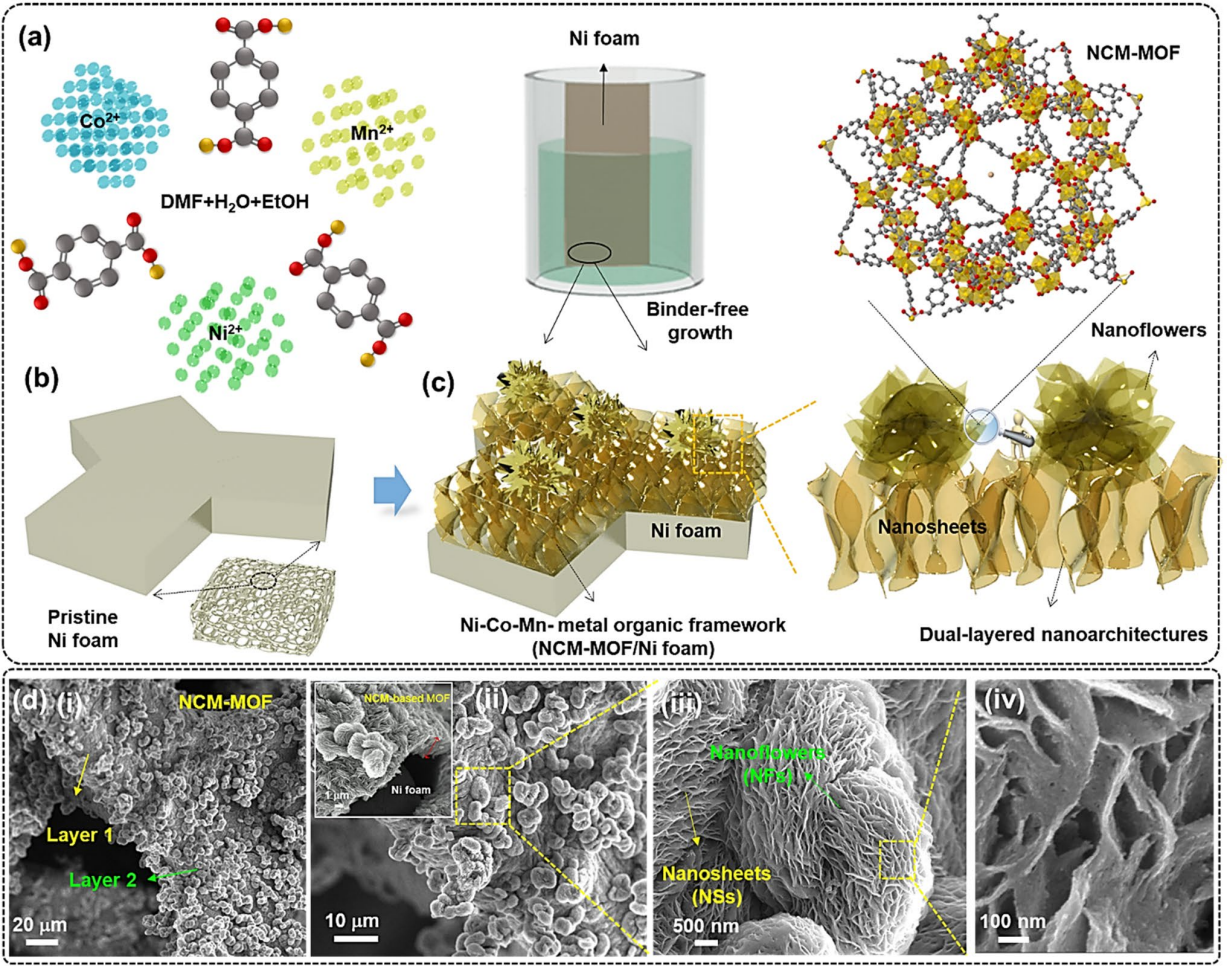
**a**–**c** A single-step growth process of the dual-layered NCM-based MOF on Ni foam using the polarity-induced solution-phased method. **d** (i–iv) Low- and high-magnification SEM images of the trimetallic NCM-based MOF-5/Ni foam, showing that the 3D flower-like nanostructures are randomly distributed on 2D nanosheets on Ni foam

An approach to decipher redox chemistry and energy storage performance of MOF-based materials is to design binder-free hierarchical nanoarchitectures with controlled geometries. The rational fabrication process of the hierarchically architectured 3D-on-2D structured Ni–Co–Mn-based trimetal–organic framework deposited on Ni foam (i.e., NCM-based MOF/Ni foam) prepared via a simple one-step solution-phase method is illustrated in Fig. 1. Herein, we chose the Ni foam as a current collector because of its multi porous property enabled by the 3D skeleton frameworks and superior conductive properties (Fig. 1b). After eradicating the native oxide layer, the Ni foam was immersed into the growth solution composed of certain amounts of Ni(NO_3_)_2_·6H_2_O, Co(NO_3_)_2_·6H_2_O, and Mn(NO_3_)_2_·6H_2_O as the metal precursors in excess amount of DMF solvent, followed by adding stoichiometric amount 1,4-bezenedicarboxylate acid as the organic ligand, respectively (Fig. 1a). To alter the polarity and increase the reaction rate, small amount of water (H_2_O) and ethanol mixture were added to the above solution and allowed it to 120 °C in hot air oven. The NCM-MOF coated Ni foam samples were collected after different growth times of 2, 5, and 10 h and the corresponding samples were labeled as NCM-based MOF-2, MOF-5, and MOF-10, respectively. Owing to the variance in the growth time, growth kinetics and nucleation rate could be varied, which led to the rationally controlled morphologies of monolayered (MOF-2), dual-layered (MOF-5), and aggregated/truncated dual-layered (MOF-10) nanoarchitectures, systematically integrated on Ni foam. The corresponding samples have the significant impact on the redox chemistry and charge storage properties when used as working electrodes. In particular, the sample synthesized with 5 h of growth time offers hierarchical morphologies for the NCM-based MOF/Ni foam, which includes initially grown 2D nanosheets, and later the 3D flower-like nanostructures were deposited, as presented in Fig. 1c.

### Morphological and Structural Characterization

The surface morphology of the prepared samples was investigated using scanning electron microscope (SEM), which provides an information of hierarchical assembled NCM-based MOF nanoarchitectures (Fig. 1d(i-iv)). With an enough growth condition (5 h), the Ni foam substrate is fully covered with rationally designed and firmly adhered NCM-based MOF nanoarchitectures, as shown in the low-magnification SEM image of Fig. 1d(i, ii). It is also visible that the as-grown NCM-based MOF consists of densely packed and interconnected 2D nanosheets with a height of 1.8 μm on Ni foam. Moreover, the self-assembled 3D nanoflowers with distinct sizes were firmly adhered over the previously grown nanosheets to form dual-layered nanoarchitectures, as presented in Fig. 1d(iii). The dual-layered 3D-on-2D NCM-based MOF/Ni foam could be expected to be used as a promising electrode for supercapatteries because it provides larger electroactive surface area, superior electrochemical activity, and wider interspace for penetration of electrolyte ions to enhance the redox reactions and charge storage properties. From the high-magnification SEM image (Fig. 1d(iv)), the 3D flower-like NCM-based MOF is also composed of a self-assembled ultrathin nanosheets (thickness: ~ 10–20 nm) with numerous nanovoids. Herein, the growth time plays a significant role in the designed construction of NCM-MOF nanostructures. During the initial stage (2 h), the NCM-based MOF with interconnected nanosheets was observed, as can be seen from the SEM image of Fig. S1a. By raising the growth time to 5 h, the nucleation rate of metal species and organic ligands was gradually increased, which results in the well-resolved self-assembly of hierarchical 3D flower-like nanostructures over 2D nanosheets with firm adherence (Fig. 1d). However, when the solution-phase growth time has been further extended to twofold (i.e., 10 h), the nanosheets/nanoflowers were agglomerated with each other to form denser and thicker nanostructures with restricted pores and voids among the dual-layered structures. In addition, the exalted growth rate of reactants under extremely high growth time leads to several surface cracks for the deposited material (Fig. S1b), which could be expected to seriously affect the rate capability during the energy storage performance.

**Fig. 2:**
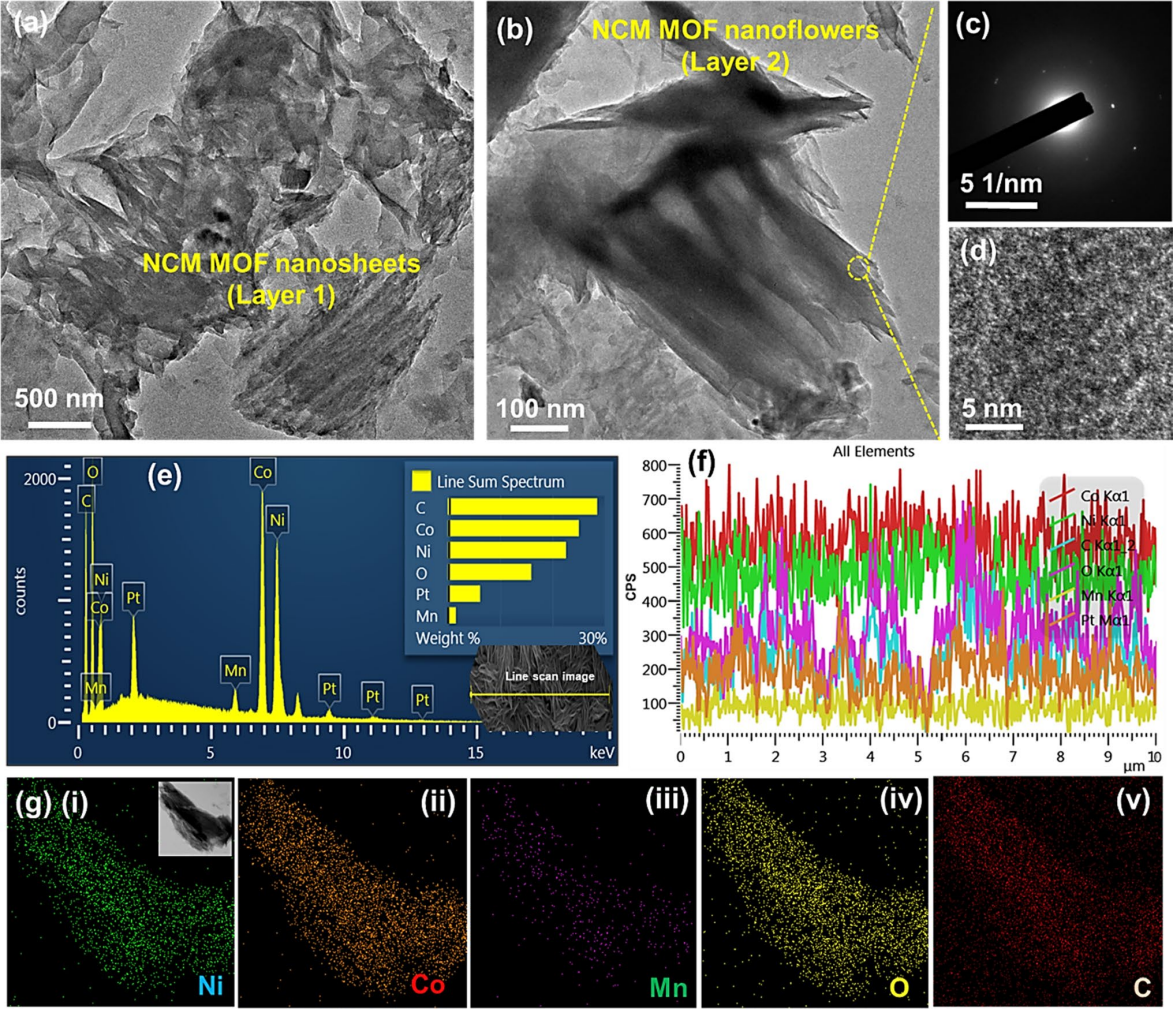
Morpholgical and elemental analysis of trimettalic NCM-based MOF peeled out from the Ni foam: TEM images of the NCM-based MOF-5/Ni foam, displaying layer 1 composed of **a** 2D nanosheets and layer 2 composed of **b** 3D flower-like nanostructures. **c** Corresponding SAED pattern and **d** HR-TEM image of the NCM-based MOF-5 nanostructures. **e** EDX spectrum, **f** line-scan EDX spectra, and **g** elemental mapping images of (i) Ni, (ii) Co, (iii) Mn, (iv) O, and (v) C elements of the NCM-based MOF-5 nanostructures

Moreover, the transmission electron microscope (TEM) analysis of NCM-based MOF nanoarchitectures has been carried to examine the structural properties of the prepared sample. For the TEM analysis, the NCM-based MOF/Ni foam was cut into small slices and ultrasonicated in ethanol to form colloidal solution of NCM nanostructures. The TEM image in Fig. 2a, b displays the NCM-based MOF hierarchical structures mainly composed of several nanosheets with interconnected assembly. After ultrasonication, the layer 1 and layer 2 were separated. The nanosheets and flower-like nanoarchitectures were clearly visible as shown in Fig. 2a, b. The selected area electron diffraction (SAED) and high-resolution TEM (HR-TEM) images in Fig. 2c, d revealed the poor diffraction patterns and lattice fringes, which may suggest the pseudo-crystallinity of the NCM-based MOF. The energy-dispersive X-ray spectroscopy (EDX) elemental analysis was carried to analyze the existed elements of the prepared sample. The EDX spectrum along with line-scan mapping data in Figs. 2e, f and S2 collectively confirms the existence of Ni, Co, Mn, C, and O elements. Moreover, the TEM elemental mapping of NCM-based MOF (Fig. 2g(i-v)) indicates the uniform distribution of Ni, Co, Mn, O, and C throughout the whole nanoarchitecture, indicating the successful formation of NCM-based MOF.

**Fig. 3:**
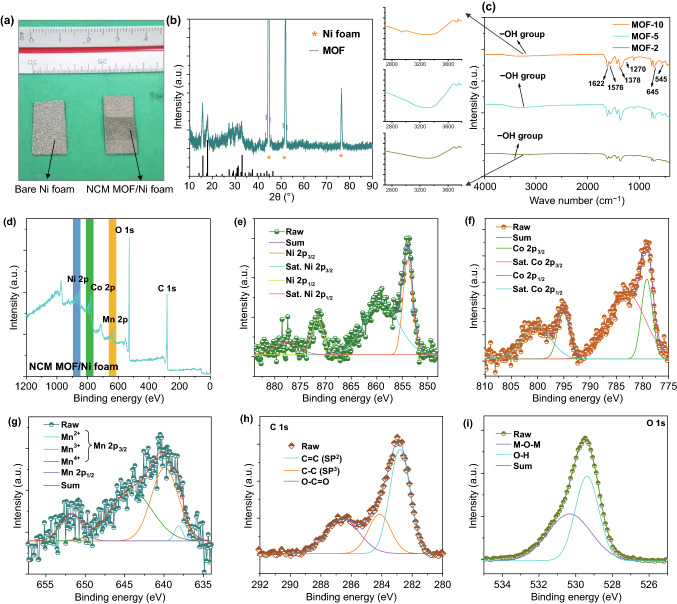
**a** Photographic image of the prepared samples, **b** XRD pattern of NCM-based MOF-5/Ni foam, **c** FT-IR spectra of MOF-NCM-based samples synthesized for various growth times. **d** XPS survey scan spectrum and high-resolution XPS spectra of **e** Ni 2*p*, **f** Co 2*p*, **g** Mn 2*p*, **h** C 1*s* and **i** O 1*s* of the dual-layered NCM-based MOF sample

The photographic images before and after the growth of NCM-based MOF on Ni foam are included in Fig. 3a. After the growth of NCM-MOF, the Ni foam color has been evidently changed to dark green, as presented in Fig. 3a. The structure, functional groups, and elemental composition of the prepared samples were further analyzed using X-ray diffraction (XRD), Fourier-transform infrared spectroscopy (FT-IR), and X-ray photoelectron spectroscopy (XPS) analyses. Figure 3b shows the typical XRD pattern of the NCM-based MOF/Ni foam electrodes. The XRD pattern of NCM-based MOF/Ni foam (5 h) revealed that the peaks are associated with the presence of NCM-based MOF and they are similar with the previously reported trimetallic MOFs [34]. These diffraction peaks indicate that the prepared MOF has a layered topology crystal structure, which was connected by central metal atoms with terephthalic acid ligands to form layered structure. Also, these layers were connected with hydrogen bonds to enable the stability of NCM-based MOF [25]. In FT-IR spectra (Fig. 3c) of the prepared samples, the broad peaks observed in 3000–3590 cm^−1^ are related to the stretching vibration of -OH groups and the absorption bands appeared at ~ 545 cm^−1^ correspond to the stretching vibration of metal–oxygen bonds. Meanwhile, XPS was utilized to explore the surface elemental compositions and chemical oxidation states of the NCM-based MOF/Ni foam (5 h sample). The typical XPS survey scan spectrum in Fig. 3d shows the characteristic peaks of Ni 2p, Co 2p, Mn 2p, O 1 s, and C 1 s in NCM-based MOF without any impurities and these results are in good agreement with the EDX and line-scan mapping analysis of Fig. 2. By using the Gaussian fitting method, the elemental XPS spectra were well fitted with spin–orbit doublets and shakeup satellites. For the Ni 2p high-resolution XPS spectra of the NCM-based MOF (Fig. 3e), the two peaks situated at 854 and 871.6 eV are related to the Ni 2p_3/2_ and Ni 2p_1/2_ spin–orbit doublets, respectively and the other two characteristic peaks of 859.6 and 877.7 eV are ascribed to the satellite (indicated by “Sat”) peaks of Ni 2p_3/2_ and Ni 2p_1/2_, respectively. These intense peaks along with satellite peaks collectively demonstrated that the most of Ni elements in the prepared NCM-based MOF are in +2-oxidation state, while the signal from +3-oxidation state of Ni is absent [35]. Similarly, the XPS spectra of Co 2p split into Co 2p_3/2_ (779.4 eV) and Co 2p_1/2_ (795.2 eV), followed by a pair of satellite bands (Fig. 3f), which indicates the high-spin Co^2+^ state in the synthesized NCM-based MOF [36]. Meanwhile, for the Mn 2p XPS spectra, the three peaks in between 638 to 644 eV related to the Mn 2p_3/2_ and the peak at 651.7 eV related to the Mn 2p_1/2_ suggest the presence of versatile oxidation states for Mn in the corresponding sample (Fig. 3g) [37]. The multicomponent metallic species with different oxidation states of the NCM-based MOF could obviously provide superior electrochemical properties during the energy storage measurements. The C 1 s spectrum (Fig. 3h) in the NCM-based MOF nanoarchitectures exhibited three peaks in between 280 and 290 eV, which are ascribed to the bonds of C = C (*sp*^2^), C–C (*sp*^3^), and O–C = O, respectively [38]. The spectrum of O 1 s exhibited two peaks at the binding energy values of 526–534 eV which correspond to the metal–oxygen-metal (M–O–M) bonds and the oxygen in –OH of the prepared sample (Fig. 3i). Also, the XPS data revealed that the contents of the elements presented in the prepared sample were observed to be 16.2, 65.22, 2.73, 10.68, and 5.17 at.% for the C 1*s*, O 1*s*, Mn 2*p*, Co 2*p*, and Ni 2*p*, respectively, in the NCM-based MOF. The corresponding XPS results further indicate the successful formation of Ni–Co–Mn terephthalate phases, respectively.

### Electrochemical Testing and Analysis

**Fig. 4:**
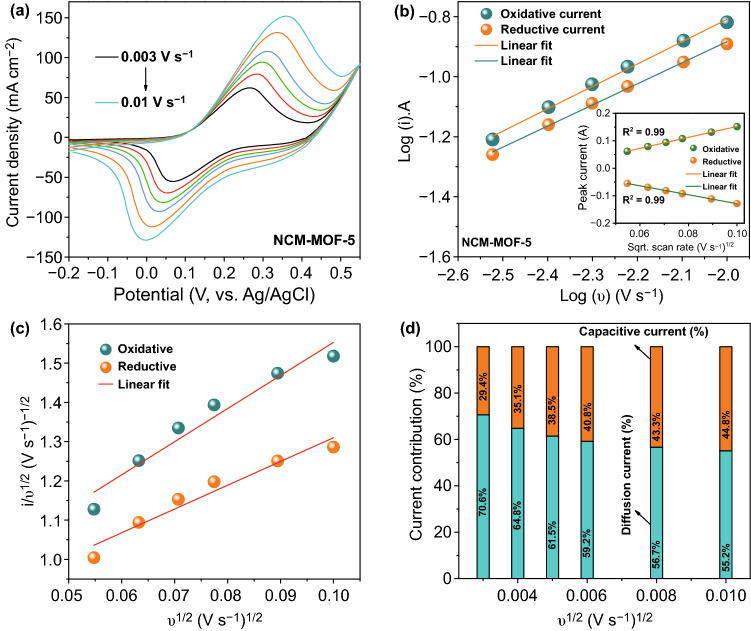
Systematic investigation on the energy storage performance of the dual-layered NCM-based MOF-5 electrode. **a** CV curves of the NCM-based MOF-5 electrode measured at different scan rates and **b** logarithm relationship of oxidation and reduction peak current versus scan rate. **c** Plots of *v*^1/2^ versus *i/v*^1/2^ used for calculating the constants of *k*_1_ and *k*_2_ with oxidative and reductive currents of CV curves. **d** Capacitive- (orange color) and diffusion-controlled (cyan color) current contribution of the NCM-based MOF-5 electrode analyzed at various scan rates. The inset in **b** shows the linear relationship of the redox peak current versus square root of scan rate

The electrochemical properties, including cyclic voltammetry (CV), galvanic charge–discharge (GCD) and electrochemical impedance spectroscopy (EIS) of the prepared samples synthesized with various growth times/morphologies (NCM-based MOF-2, MOF-5, and MOF-10, respectively) were measured in a three-electrode cell with an aqueous 1 M KOH electrolyte solution at room temperature, as presented in Fig. 4. The CV curves of the NCM-based MOF-5 at various scan rates ranging from 0.003 to 0.01 V s^−1^ are shown in Fig. 4a. From the CV curves, the NCM-based MOF-5 showed dominant oxidation and reduction peaks during the anodic and cathodic sweeps, which are explicitly observed for each scan rate. Moreover, the redox peak currents were exalted with respect to the increased scan rate, which reveals that the redox peak currents depend on the scan rate. In addition, it is visible that the peak potentials shift to the positive and negative sides under the increased scan rate, indicating the quasi-reversibility of the material, and it is mainly due to the ohmic resistance/low ionic diffusivity of the redox-type active materials. From the existed redox peaks in CV curves, it is worth mentioning that the NCM-based MOF-5 showed the typical battery-type charge storage behavior due to the redox transitions of Ni^2+^/Ni^3+^, Co^2+^/Co^3+^, and Mn^3+^/Mn^4+^ in the NCM-based MOF with an aid of hydroxyl (OH^−^) ions, respectively. However, to verify the contribution of charge storage mechanisms, the classification of capacitive/diffusion-controlled energy storage contributions is explained as follows. Generally, the redox reactions can be observed in electrochemically active metal-based materials [i.e., (1) pseudocapacitive redox behavior (in MnO_2_) and (2) diffusion-controlled redox behavior (in NiO, MoO_3_)] and the non-Faradaic behavior is attributed from the carbon-based materials [12, 39–41]. Among these charge storage processes, diffusion-controlled energy storage materials offer superior charge storage performance compared to the other class of materials. To quantify the charge storage behavior of the NCM-based MOF-5 explicitly, the power’s law and modified power’s law were utilized. As per the power’s law equation: *i* = *aν*^*b*^, where *a* and *b* are adjustable parameters and *ν* is the applied scan rate, respectively. For this analysis, the logarithm was applied to the both ends of the formula and it can be mentioned as [42–44]:

1 $$\log \, i \, = \log \, a\nu^{b} = \, \log \, a \, + \, b \, \log \nu$$

2 $$b = \frac{\log i - \log a}{\log } = \frac{\log i}{\log } - \, \log_{\nu } a \, \left( {\text{constant}} \right)$$

From this formula, the expected “*b*” value in between 0.5 to 1 can be calculated from the slope of log (i) versus log (*ν*) plot. Regarding the kinetic surface-controlled charge storage mechanism (i.e, capacitive-type), the b value is approximate to 1, while it is around 0.5 when there is an existence of diffusion-dominated course of charge storage within the active materials. As obtained from Fig. 4b, the resulted b values for the NCM-based MOF-5 electrode during the oxidation and reduction processes were about 0.68 and 0.7, which indicates the diffusion-controlled and/or mixed charge storage behavior of the material. To quantify the maximum amount of charge stored by the capacitive-type (k_1_*ν*) and battery-type diffusion-controlled (*k*_2_*ν*^1*/2*^) mechanisms in the peak regions, the power’s law could be modified into the following equations [19]:

3 $$i = k_{1} \nu + k_{2} \nu^{1/2}$$

And it can be altered into

4 $$i/\nu^{1/2} = \, k_{1} \nu^{1/2} + k_{2}$$

where *i*, *ν, k*_1_, and *k*_*2*_ indicate the current, scan rate, and constants, respectively. By plotting the linear fit of *i/ν*^1/2^ versus *ν*^1/2^ (Fig. 4c), the slope and intercept values of k_1_ and k_2_ can be obtained. Thus, the portion of capacitive- and diffusion-controlled charge storage contributions was calculated using Eq. (3) and the obtained results are plotted in Fig. 4d. The contribution ratios of battery-type current were 70.6, 64.8, 61.5, 59.2, 56.7, and 55.2% at the scan rates of 0.003, 0.004, 0.005, 0.006, 0.008, and 0.01 V s^−1^, respectively, suggesting that the capacitive-type contribution is relatively lower for the NCM-based MOF-5. Hence, the diffusion-controlled capacity plays a decisive role in the charge storage performance of the NCM-based MOF-5 material. This can be further confirmed by the plots of oxidative and reductive currents with respect to the scan rate, showing the perfect linearity (R^2^ = 0.99). This indicates that the redox chemistry in dual-layered NCM-based MOF-5 was facilitated by the deep penetration of OH^−^ ions (inset of Fig. 4b), which instructs the diffusion-controlled charge storage mechanism of the corresponding material.

**Fig. 5:**
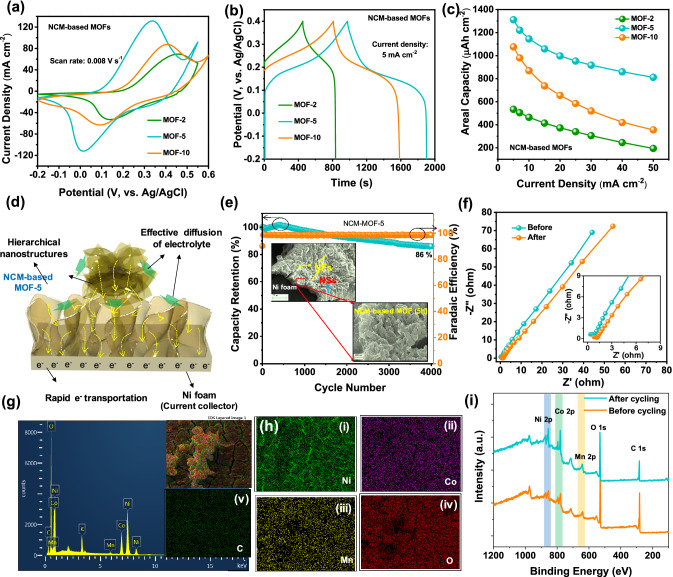
Comparitive **a** CV curves evaluated at constant scan rate of 0.008 V s^−1^, **b** GCD curves measured at a current density of 5 mA cm^−2^, and **c** estimated capacity values of the NCM-based MOF-2, MOF-5, and MOF-10 electrodes, respectively. **d** Schematic illustration regarding the merits of the prepared electrodes during electrochemical measurements. **e** Cyling stability of the NCM-based MOF-5 electrode over 4000 charge–discharge cycles and **f** EIS plots of the NCM-based MOF-5 electrode before and after the cycling process. The inset of **e** shows the FE-SEM images of NCM-based MOF-5 after the cycling test. **g** EDX spectrum, **h** elemental mapping images, and **i** XPS survey scan spectra of NCM-based MOF-5 after the cycling test, which indicates that the respective elements still existed on the NCM-based MOF-5 material

The effect of growth time on the electrochemical performance of the NCM-based MOF materials was compared, as shown in the CV (measured with a constant scan rate of 0.008 V s^−1^) and GCD curves (measured with a current density of 5 mA cm^−2^) of Fig. 5a, b. The redox behavior in the NCM-based monolayered MOF-2, dual-layered MOF-5, and truncated MOF-10 nanoarchitectured electrodes clarifies the battery-type mechanism of the materials. As compared to the NCM-based monolayered MOF-2 and truncated MOF-10 electrodes, the dual-layered NCM-based MOF-5 demonstrated superior redox chemistry, owing to the multidimensional structures, numerous nanovoids, and rich redox sites of guest species (Ni, Co, and Mn species). From the comparative GCD curves, it is also evident that the dual-layered NCM-based MOF-5 shows higher charge–discharge times, which further reveals the excellent capacity of electrode material. The mass of the deposited material varies under different growth times, which affects the gravimetric capacity, and thus the areal capacity values were calculated for the synthesized samples. The CV and GCD curves of the corresponding samples at various scan rates and current densities are included in Figs. S3–S5. The estimated areal capacity values from the GCD curves of these electrodes were plotted for the applied current densities, using the following Eq. (5) and compared in Fig. 5c [45, 46]:

5 $$Q_{ac} = \frac{I \times \Delta t}{a}$$

where ‘*Q*_ac_’ is the areal capacity (Ah cm^-2^), ‘*I*’ is the discharge current (A), ‘*∆t*’ is the discharge time (s), and ‘*a*’ is the area of the electrode active area (cm^2^), respectively. At a current density of 5 mA cm^−2^, the capacity values of the NCM-based MOF-2, MOF-5, and MOF-10 were 533.5, 1311.4, and 1075.9 μAh cm^−2^, respectively. Also, the dual-layered NCM-based MOF-5 showed a superior rate capability of 61.8% (811.67 μAh cm^−2^), compared to the monolayered MOF-2 of 31.8% (194.5 μAh cm^−2^) and MOF-10 of 33% (355.27 μAh cm^−2^) at the tenfold increased current density (50 mA cm^−2^). The increased capacity and rate capability of the dual-layered NCM-based MOF-5 as compared with the monolayered MOF-2 and truncated MOF-10 were schematically conjucted in Figs. 5d and S6. The monolayered NCM-based MOF-2 provides the paths for electrolyte diffusion owing to the interconnected and porous property of nanosheets (Fig. S6a). However, the monolayered material could not show better redox chemistry owing to the moderate mass of the deposited material. Meanwhile, the dual-layered NCM-based MOF-5 is composed of 3D-on-2D nanoarchitectures, from which the 2D nanosheets of NCM-based MOF not only enhance the redox active sites but also act as an electron superhighway to remove the interfacial resistance, as presented in Fig. 5d. In addition, the 3D nanoflowers of NCM-MOF further enhance the electroactive surface area and offer more redox active sites for superior energy storage. On the other hand, the NCM-based MOF-10 showed lower capacity than the MOF-5 sample, which could be due to the hindered paths for electrolyte diffusion on the truncated/agglomerated morphology and the decreased rate capability is due to the thicker/surface cracks of the material (Fig. S6b). It is also worth mentioning that the capacity of our dual-layered NCM-based MOF-5 electrodes is comparatively higher than the previous works, as included in Table S1. The cycling stability is another important factor, which determines the electrode suitability for practical applications. Therefore, the cycling stability of the NCM-based MOF-5 sample was measured for 4000 cycles with a current density of 25 mA cm^−2^. After the long-term cycling process, the material demonstrated good cycling stability of 86% with a good Faradaic efficiency of 98.4%, as presented in Fig. 5e. Even after the cycling test, the material retained its good structural stability without degrading, as shown in the inset of Fig. 5e. The elemental analysis and composition of the cycled electrode were examined using EDX and XPS analyses, as presented in Fig. 5g–i. As shown in the EDX spectrum of Fig. 5g, the elements including Ni, Co, Mn, O, C, and K were presented. The peak of K is due to the KOH electrolyte. Also, the corresponding elemental mapping images also illustrate that the NCM-based MOF showed strong durability even after the cycling test. The XPS peaks in the survey scan spectra further confirm that all the elements existed in the sample before and after the cycling test. The good cycling stability could be ascribed to the ternary component fabricated in a single approach with the hierarchical self-structured bilayer arrangement. The EIS analysis of the NCM-based MOF-5 electrode was performed before and after the cycling test (Fig. 5f), which indicates the common features of negligible semi-circle in the high-frequency region and the slanted straight line in the low-frequency region, which leads to the charge transfer resistance (*R*_ct_) and Warburg impedance (Z_w_), respectively. Meanwhile, the intercept at the real axis in the high-frequency region shows internal resistance (*R*_s_) which mainly originates from the electrode or ionic resistance of electrolyte. Even after the cycling test, the material showed a good *R*_ct_ value of 0.52 Ω which is close to the *R*_ct_ of 0.4 Ω for the fresh sample, referring to the good conductivity and excellent electrochemical kinetics of the dual-layered NCM-based MOF-5. These interesting electrochemical results along with good cycling stability could serve as an effective battery-type electrode for supercapatteries.

### Fabrication and Electrochemical Testing of Supercapattery

**Fig. 6:**
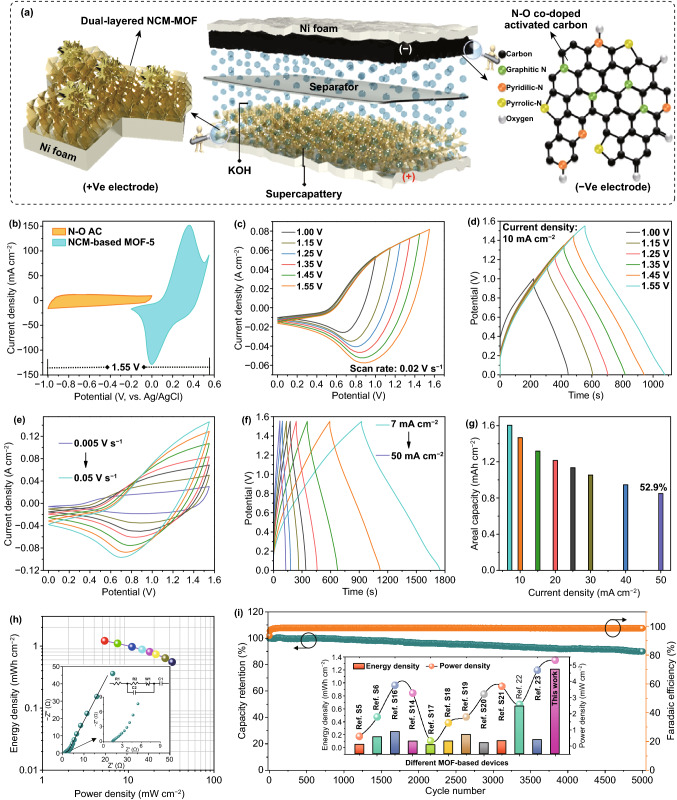
**a** Schematic illustration showing the fabrication of supercapttery device and **b** CV curves of the dual-layered NCM-based MOF-5 and biomass-derived N–O AC electrodes measured individually at a scan rate of 0.01 V s^−1^, indicating the possible cell potential of 1.55 V. **c** CV and **d** GCD curves of NCM-based MOF-5//N–O AC device measured at various potential windows of 0–1.0  to 0–1.55 V. **e** CV curves and **f** GCD curves of the supercapattery system measured at various scan rates and current densities of 0.005–0.05 V s^−1^ and 7–50 mA cm^−2^, respectively. **g** Calculated areal capacity response with respect to the applied discharge current density. **h** Ragone plot and **i** cycling stability of the supercapttery. The insets in **h** and **i** show the Nyquist plot of the fabricated device and comparative energy and power densities of various supercapattery devices with previous works, respectively

An effective demonstration of the dual-layered NCM-based MOF-5 as a battery-type electrode along with the nitrogen–oxygen-enriched activated carbon (N–O AC) as a negative electrode results in a curious hybrid paradigm, so-called, supercapatteries that could help to meet the needs of improved energy storage performance. The both electrodes in supercapattery system was separated with a cellulose filter paper (Whatman^®^) and few ml of an aqueous alkaline electrolyte (1 M KOH) was used as an electrolyte, as displayed in the schematic illustration of fabricated supercapattery (Fig. 6a). Herein, N–O AC was prepared using a squid fish biomass and it was treated by alkali activation and pyrolysis of squid fish [47]. Based on the potential window of the NCM-based MOF-5 (− 0.2 to 0.55 V) and N–O AC (− 1 to 0 V), the operating potential of the supercapattery system is projected to 0–1.55 V, as shown in Fig. 6b. To further examine the suitable operating potential window, the fabricated supercapattery was tested with different cell potentials using CV and GCD analyses. Figure 6c, d shows the CV and GCD curves of the NCM-based MOF-5//N–O AC device under different cell potentials. It is noteworthy that even at high cell potential range (0–1.55 V), the CV and GCD curves of supercapattery showed no obvious polarization and mainly displayed the mixed energy storage mechanisms of battery-type (due to redox active sites deciphered NCM-based MOF-5 nanostructures) and capacitive-type (initiating from surface absorption of ions on N–O AC) in the assembled device. Therefore, the cell potential was fixed to 0–1.55 V and the CV and GCD analyses were performed at various scan rates and current densities. Figure 6e displays the CV curves of the NCM-based MOF-5//N–O AC device at the scan rates of 0.005–0.05 V s^−1^ with an operating potential window of 0–1.55 V. Even at high scan rates, the shape of the device can remain the same and the obvious redox peaks along with capacitive characteristics of supercapattery indicate the good reversibility and electrochemical kinetics of the device. The GCD analysis of the fabricated device was further carried out to analyze the energy storage properties, as shown in Fig. 6f. The potential versus time profile and charge–discharge phenomenon of the GCD curves collectively assess the superior degree of reversibility and efficiency, respectively. The calculated areal capacities of supercapattery device using the discharge times were 1.6, 1.46, 1.31, 1.2, 1.13, 1.05, 0.94, and 0.84 mAh cm^−2^ at the current densities of 7, 10, 15, 20, 25, 30, 40, and 50 mA cm^−2^, respectively, as displayed in Fig. 6g. The excellent rate capability of 52.9% at higher current density of 50 mA cm^−2^ further demonstrates the unique mass balancing and good charge storage properties of the fabricated supercapattery. As important parameters to demonstrate the energy storage performance of supercapattery system for real-time applications, the energy density (*E*_d_) and power density (*P*_d_) values of the NCM-based MOF-5//N–O AC device were calculated according to the following formulae [13, 14]:

6 $$E_{\text{d}} = \frac{I \times \smallint V\left( t \right)dt}{a \times 3.6}$$

7 $$P_{\text{d}} = \frac{{E_{\text{d}} }}{\Delta t} \times 3600$$

where *I* is the discharge current (A), *∆t* is the discharge time (s), *a* is the working area of the device (cm^2^), and $\smallint V\left( t \right)dt$ is the area under the discharge curve, respectively. The Ragone chart (energy density versus power density) of the assembled supercapattery is included in Fig. 6h. Remarkably, the device delivered a maximum energy density of 1.21 mWh cm^−2^ at a power density of 5.3 mW cm^−2^ and still keeps 0.55 mWh cm^−2^ at a high power density of 32.49 mW cm^−2^, respectively. Obviously, the energy density of the NCM-based MOF-5//N–O AC supercapattery is much higher than that of the metal oxide/sulfide-based hybrid supercapacitors, such as nanoporous Co_3_O_4_//AC (0.072 mWh cm^−2^ at 3.2 mW cm^−2^) [48], core–shell-like CoMoO_4_@Co(OH)_2_//porous AC (0.167 mWh cm^−2^ at 1.5 mW cm^−2^) [49], Ni-Co-S//AC (0.1 mWh cm^−2^ at 3.27 mW cm^−2^) [50], nanocomposite Ni_3_S_2_@CdS//carbon (0.26 mWh cm^−2^ at 1.48 mW cm^−2^) [51], etc. Moreover, the NCM-based MOF-5//N–O AC supercapattery showed superior/comparable energy storage performance to many other solitary and binary MOF-based hybrid devices, including Ni-MOF//AC (0.046 mWh cm^−2^ at 0.6 mW cm^−2^) [52], CNT@Ni-MOF//rGO (0.17 mWh cm^−2^ at 1.8 mW cm^−2^), mixed Ni-Co-MOF//AC (0.64 mWh cm^−2^ at 2.57 mW cm^−2^) [32], Co-Mn MOFs//AC (0.12 mWh cm^−2^ at 4.68 mW cm^−2^) [33], etc. The detailed comparative results of our supercapattery with many other previous reports are included in the inset of Fig. 6i and Table S2. The superior charge storage performance of our device is mainly ascribed to an exalted redox chemistry of the dual-layered NCM-based MOF-5 and variation in electronegativity from the hetero atoms doping in AC (N–O AC), which enables the superior capacitive-properties. The long-term durability of the fabricated supercapattery was further tested to evaluate the electrochemical durability upon continuous GCD cycles. The cycling stability of the NCM-based MOF-5//N–O AC supercapattery was measured at a high current density of 30 mA cm^−2^ for 5000 cycles, as presented in Fig. 6i. The capacity of supercapattery retains 90.1% along with good Faradaic efficiency of 98.6%, demonstrating the good cycling durability of the device. The fitted EIS plot of the corresponding device with equivalent circuit demonstrates the lower *R*_ct_ of the device, indicating the good ionic as well as electrochemical kinetics of the device, as presented in the inset of Fig. 6h. Overall, the proposed dual-layered NCM-based MOF-5//N–O AC device and its obtained energy storage properties pave a new method for gaining superior energy storage for practical applications.

### Self-powered Renewable Energy Storage/Conversion Applications

**Fig. 7:**
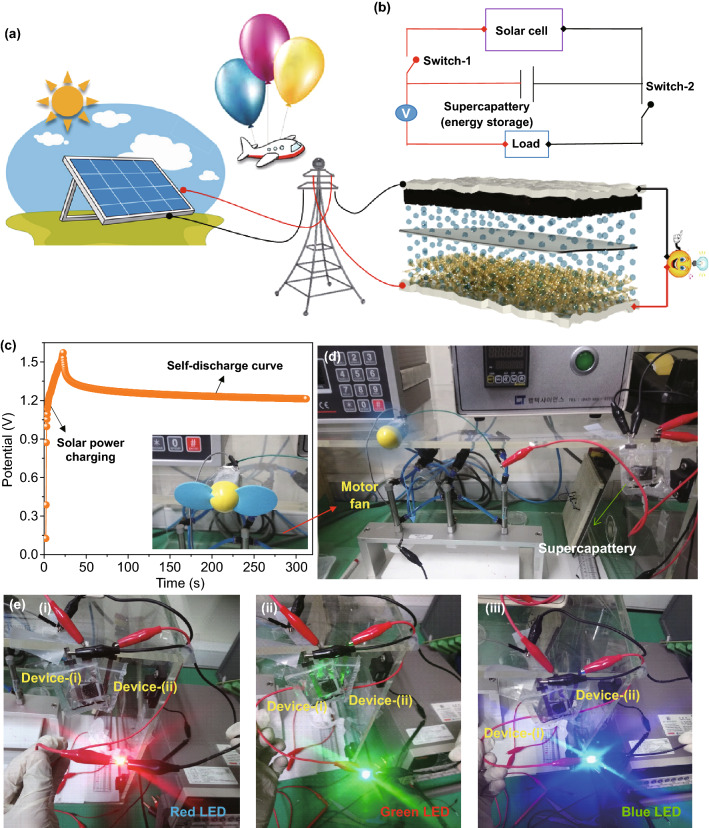
**a** Schematic illustration of the self-powered charging station with integrated solar energy driven supercapttery devices. **b** Circuit diagram and **c** charging and self-discharge curves of solar energy-based supercapttery. **d** Working condition of a motor fan and **e** powering of various colored LEDs with solar energy-assisted supercapattery

Adding to the high-energy-storage properties, the MOF-based supercapattery system was further utilized to harvest the solar power energy for self-powered electronic applications. As we know, the sunlight energy is one of vital renewable resources and such energy can be captured and transformed into electricity during the fall of sunlight on corresponding harvesting devices. Harvesting the solar energy with an effective energy storage system could effectively meet the future energy challenge based on increasing human civilization. Herein, we attempted to fabricate a proto-type solar-driven renewable NCM-based MOF-5//N–O AC supercapattery system, as shown in Fig. 7a. Figure 7b shows the circuit diagram for solar cell and two serially connected supercapattery stems. When the solar light is incident on the solar cell, it generated green electricity, which can be effectively used to charge supercapattery device within a short period, as shown in the charging curve of Fig. 7c. When the solar cell was disconnected, the supercapattery holds its potential for long time during the self-discharging process, indicating the device applicability for myriad portable electronics. By exploiting the high energy density of the solar power-driven supercapattery, the device successfully energized a toy motor fan, as presented in Fig. 7d. Moreover, two serially connected supercapatteries were also able to glow various colored light-emitting diodes (LEDs) for long time, as presented in Fig. 7e. The solitary supercapattery also energized a multifunction electronic display, as presented in Fig. S4, which reveals great potential of the solar power-driven supercapattery-based on NCM-based MOF-5 battery-type electrode for use in various energy applications.

## Conclusion

In summary, we developed controlled morphologies of binder-free NCM-based MOFs by the polarity-induced solution-phased method for high-performance supercapattery applications. With an optimal growth condition, the dual-layered NCM-based MOF with 3D-on-2D nanoarhitectures was rationally integrated on Ni foam with good adhesion. The multifunctional properties of dual-layered NCM-based MOFs showed a maximum capacity of 1311.4 μAh cm^−2^ at a current density of 5 mA cm^−2^ with good cycling stability of 86.1% in aqueous alkaline electrolyte. The enhanced energy storage performance of dual-layered NCM-based MOFs is mainly due to the synergistic features of highly accessible active area and exalted redox chemistry of guest species (Ni, Co, and Mn). Adding to the advantage of high capacity, the dual-layered NCM-based MOFs were further employed as a battery-type electrode with the biomass-derived N–O AC as a negative electrode into a pouch-type supercapattery assembly, which showed superior energy and power densities of 1.21 mWh cm^−2^ and 32.49 mW cm^−2^, respectively. Utilizing high-energy storage properties, the NCM-based MOF-5//N–O AC device was integrated with a solar power system for self-powered portable electronic applications. With the superior redox chemistry and higher energy storage properties along with renewable energy harvesting/storage abilities, this device will further stimulate the widespread attention to the development of hierarchical MOF-based nanostructures for potential energy storage devices.

## Experimental Details

### Materials

The starting materials of nickel nitrate hexahydrate (Ni(NO_3_)_2_·6H_2_O), cobalt nitrate hexahydrate (Co(NO_3_)_2_·6H_2_O), manganese nitrate tetrahydrate (Mn(NO_3_)_2_·6H_2_O), and 1,4‐bezenedicarboxylate acid (1,4‐BDC, C_8_H_6_O_4_) were purchased from Sigma-Aldrich (South Korea). N,N-Dimethylformamide (DMF), KOH, hydrochloric acid (HCl), and ethanol were purchased from Daejung Chemicals, Ltd., (South Korea). The substrate Ni foam was obtained from MTI Korea. All the reagents used in the study are of analytical grade and used as received without further purification.

### Preparation of Trimetallic Ni–Co–Mn-based MOFs on Ni Foam

The dual-layered trimetallic NCM-based MOF nanoarchitectures were synthesized on Ni foam by a facile polarity-induced solvothermal method under controlled growth parameters. Prior to the growth process, Ni foam slices (1 × 2 cm^2^) were ultrasonicated in 1 M HCl to remove the native oxide layer and rinsed with de-ionized (DI) water for several times, respectively. Meanwhile, an oven-dried Teflon liner was charged with Ni(NO_3_)_2_·6H_2_O, (0.75 g, 2.6 mmol), Co(NO_3_)_2_·6H_2_O (0.75 g, 2.6 mmol), Mn(NO_3_)_2_·6H_2_O (0.25 g, 1 mmol) and 1,4‐bezenedicarboxylate acid (0.49 g, 3 mmol) in an N_2_-flowed mixture of DMF/ethanol/DI water (4:1:1) and vigorously stirred at room temperature (RT) for 20 min. After that, the Ni foam samples were immersed into the above solution, transferred into an autoclave system, and heated at 120 °C for different growth intervals of 2, 5, and 10 h, respectively. Upon completion, the autoclave was cooled to RT and the MOFs coated Ni foam samples were washed with ethanol and DI water, followed by drying at 60 °C. After the growth of NCM-based MOF, the color change of Ni foam could clearly indicate the successful growth of the corresponding sample (Fig. 3a). The samples prepared under different growth times were labeled as NCM-based MOF-2 (8.2 mg), MOF-5 (11 mg), and MOF-10 (12.7 mg), respectively.

### Characterization

SEM (Carl Zeiss, LEO SUPRA 55, 5 kV) and TEM (JEM 200CX, JEOL, 200 kV) equipped with the EDX measurements were used to characterize the surface morphology and elemental mapping images of the prepared samples. XRD (M18XHF-SRA, Mac Science) and XPS (Thermo Electron MultiLab 2000) were used to characterize the structure and surface chemical composition/chemical states of the elements in the samples.

### Electrochemical Measurements

All the electrochemical measurements were performed with an Iviumstat electrochemical workstation (The Netherlands). Prior to the electrochemical measurements, the samples were soaked in 1 M KOH solution for 1 h. The electrochemical properties, using CV, GCD, and EIS (frequency range: 0.01 Hz to 100 kHz with 5 mV amplitude) analyses, were measured with a three-electrode cell composed of NCM-based MOFs, Pt wire, and Ag/AgCl electrode as the working, counter, and reference electrodes, respectively, in aqueous 1 M KOH electrolyte. The biomass-derived N–O AC was coated on Ni foam with the mixing of N–O AC with super P carbon black and polyvinylidene fluoride as the binder dissolved in N-methyl-2-pyrrolidone solvent in a weight ratio of 80:10:10. The resulting slurry was brush coated onto the Ni foam (1 × 1 cm^2^) and dried at 80 °C for 5 h in a vacuum oven. Since the NCM-based MOFs showed different mass loadings under various growth timings, we calculated the areal capacity instead of gravimetric capacity. The areal capacity of the prepared materials was calculated using the following formulae [49]:

8 $$Q_{\text{ac}} = \frac{I\times\Delta t}{a}$$

9 $$C_{\text{ac}} = \frac{I \times\Delta t}{\Delta V \times a}$$

where ‘*Q*_*ac*_*’* is the areal capacity (Ah cm^−2^), ‘*C*_*ac*_*’* is the areal capacitance (F cm^−2^) ‘*I*’ is the discharge current (A), ‘*∆t*’ is the discharge time (s), ‘*a*’ is the area of the electrode active area (cm^2^) and ‘*∆V*’ is the potential window (V).

### Fabrication of Pouch-Type Supercapattery

A two-electrode system-based pouch-type supercapattery was fabricated by a battery-type bilayered NCM-based MOF-5/Ni foam as a positive (cathode) electrode and capacitive-type N–O AC/Ni foam as a negative (anode) electrode in 1 M KOH electrolyte. A piece of filter paper was placed between the both electrodes to restrict short-circuits during the device fabrication. Then, the sandwich assembly was sealed with a pouch-like plastic bag using a commercial grade heat sealer. To balance the charges on both the electrodes, the mass ratio of the positive electrode (NCM-based MOF-5/Ni foam) to the negative electrode (N–O AC) was optimized using the following formula:

10 $$\frac{{m^{ + } }}{{m^{ - } }} = \frac{{C_{ac}^{ - } \Delta V^{ - } }}{{Q_{ac}^{ + } }}$$

where *m*^+^ is the mass and Q_ac_^+^ is the areal capacity of the positive electrode, and ∆V^−^, m^−^, and C_ac_^−^ are the potential window (V), mass (g), and areal capacitance (F cm^−2^) of the negative electrode, respectively. Based on the above formula, the optimal mass ratio of the positive electrode to the negative electrode was tuned by a series of trails and finally fixed to 0.44 in the device. Subsequently, the areal energy and power densities of the fabricated supercapattery were estimated using the following formulae [14, 53]:

11 $$E_{\text{d}} = \frac{I \times \smallint V\left( t \right)dt}{a}$$

12 $$P_{\text{d}} = \frac{{ E_{\text{d}} }}{\Delta t}$$

where *‘E*_*d*_*’* is the areal energy density (Wh cm^−2^), $\smallint V\left( t \right)dt$ is the area under the discharge curve, *‘P*_*d*_*’* is the areal power density (W cm^−2^), and ‘*∆t*’ is the discharge time (s), respectively.

### Fabrication of Self-powered Solar Power//Supercapattery System

For the fabrication of solar power storage self-powered unit, the pouch-type solitary and serially connected supercapatteries were attached to a commercially purchased solar cell via electrical switches, as demonstrated in Fig. 7a. Under the sun light, the solar cell gives electricity and charges the supercapattery effectively. The charge–discharge processes were reordered with Keithley 6514 electrometer.

## Electronic supplementary material

Below is the link to the electronic supplementary material.

ESM includes photographs of bare and NCM-based MOF coated Ni foam, SEM images of NCM-based MOF-2/Ni foam and NCM-based MOF-10/Ni foam electrodes, EDX spectrum and line mapping images, electrochemical properties of MOF-based NCM-2, GCD curves of MOF-based NCM-5, electrochemical properties of MOF-based NCM-10, and comparison of energy storage performance of NCM-based MOF in three- and two-electrode systems (PDF 1197 kb)
