# Supplementary material for: Ternary MOF-Based Redox Active Sites Enabled 3D-on-2D Nanoarchitectured Battery-Type Electrodes for High-Energy-Density Supercapatteries
Source: Nanomicro Lett. 2020 Nov 2;13:17. doi: 10.1007/s40820-020-00528-9 (PMC8187485; doi:10.1007/s40820-020-00528-9)
Supplement: Supplementary file 1 — ESM includes photographs of bare and NCM-based MOF coated Ni foam, SEM images of NCM-based MOF-2/Ni foam and NCM-based MOF-10/Ni foam electrodes, EDX spectrum and line mapping images, electrochemical properties of MOF-based NCM-2, GCD curves of MOF-based NCM-5, electrochemical properties of MOF-based NCM-10, and comparison of energy storage performance of NCM-based MOF in three- and two-electrode systems (PDF 1197 kb) [file 40820_2020_528_MOESM1_ESM.pdf]

Supporting Information for

Ternary MOFs-Based Redox Active Sites Enabled 3D-on-2D Nanoarchitectured Battery-Type Electrodes for High-Energy Density Supercapatteries

Goli Nagaraju[1, 2], S. Chandra Sekhar[1], Bhimanaboina Ramulu[1], Sk. Khaja Hussain[1, 2], D. Narsimulu[1], Jae Su Yu[1, *]

[1]Institute for Wearable Convergence Electonics, Department of Electronic Engineering, Kyung Hee University, 1732 Deogyeong-daero, Yongin-si, Gyeonggi-do 17104, Republic of Korea

[2]Department of Chemical Engineering, College of Engineering, Kyung Hee University, 1732 Deogyeong-daero, Gihung-gu, Yongin-si, Gyeonggi-do 17104, Republic of Korea

*Corresponding author. E-mail: jsyu@khu.ac.kr (Jae Su Yu)

Supplementary Figures and Tables

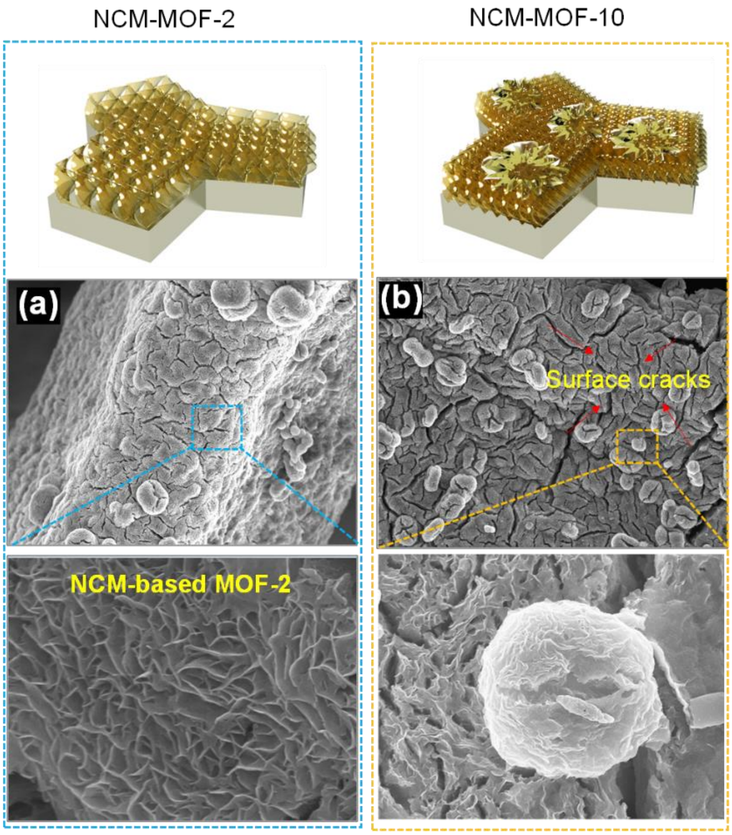

Fig. S1 Low- and high-magnification SEM images of the (**a**) NCM-based MOF-2/Ni foam and (**b**) NCM-based MOF-10/Ni foam electrodes

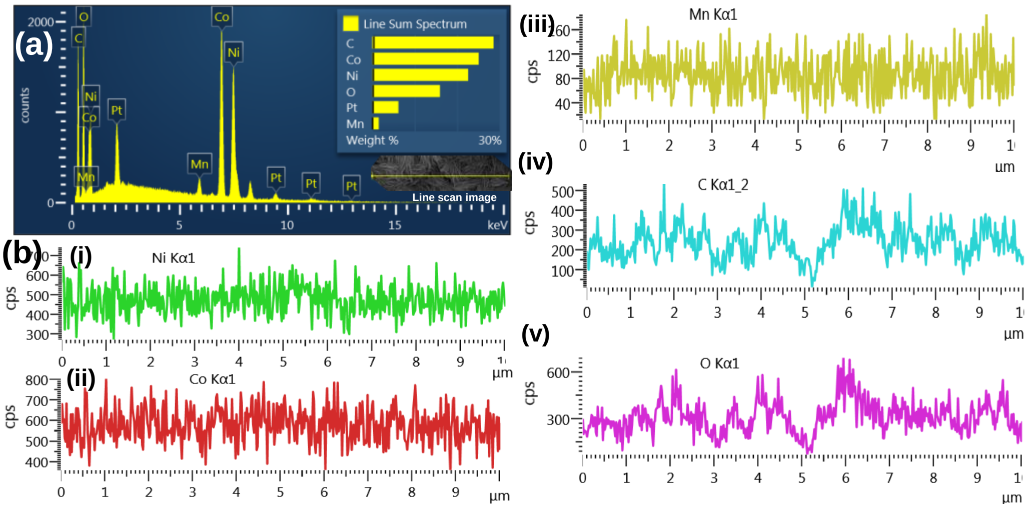

Fig. S2 EDX spectrum and line-scan spectra of individual elements (Ni, Co, Mn, C and O) for the NCM-based MOF-5/Ni foam

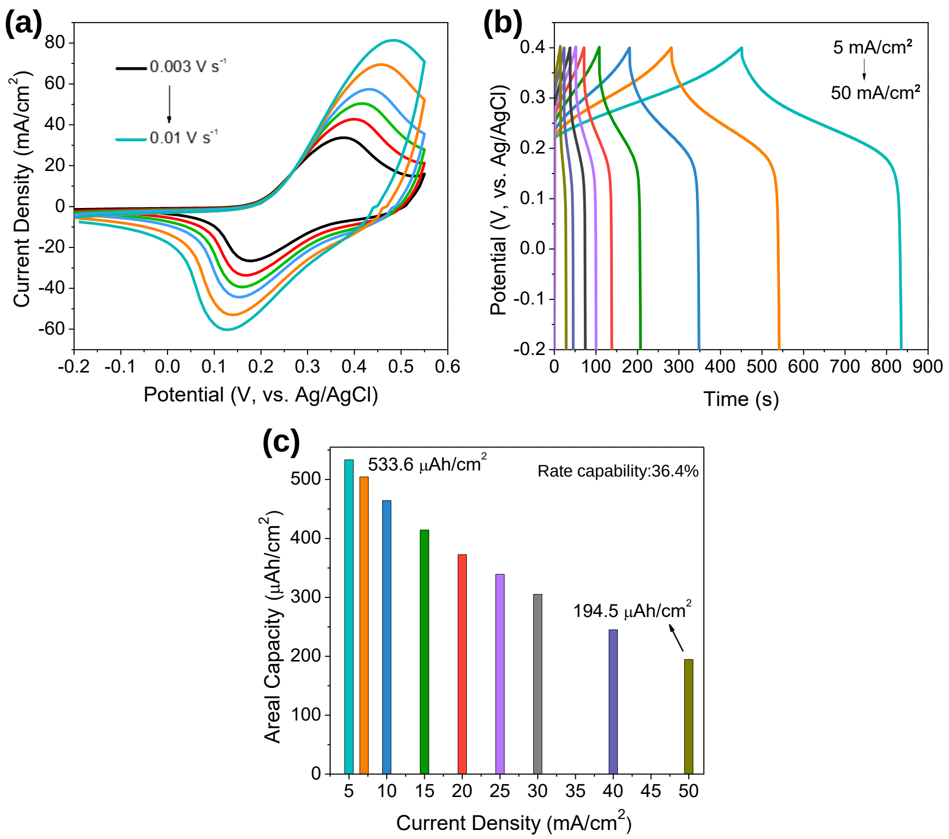

Fig. S3 Electrochemical properties of the monolayered MOF-based NCM-2 tested in 1 M KOH electrolyte. (**a**) CV curves evaluated at different scan rates of 0.003-0.01 V s^{-1}, (**b**) GCD curves measured at different current densities of 5-10 mA cm^{-2} and (**c**) estimated capacity values as a function of current density for the monolayered MOF-based NCM-2

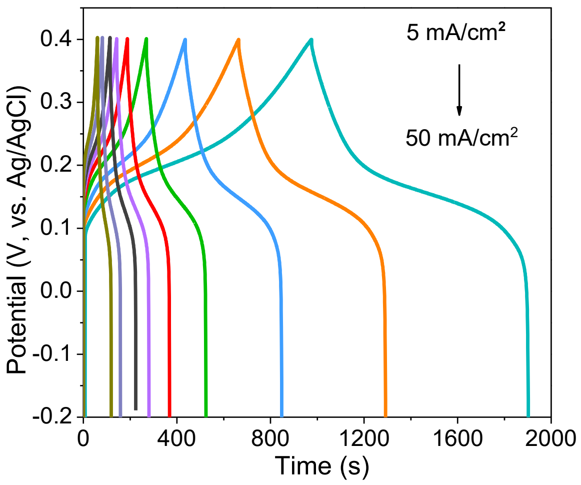

Fig. S4 GCD curves measured at different current densities of 5-10 mA cm^{-2} for the dual layered MOF-based NCM-5

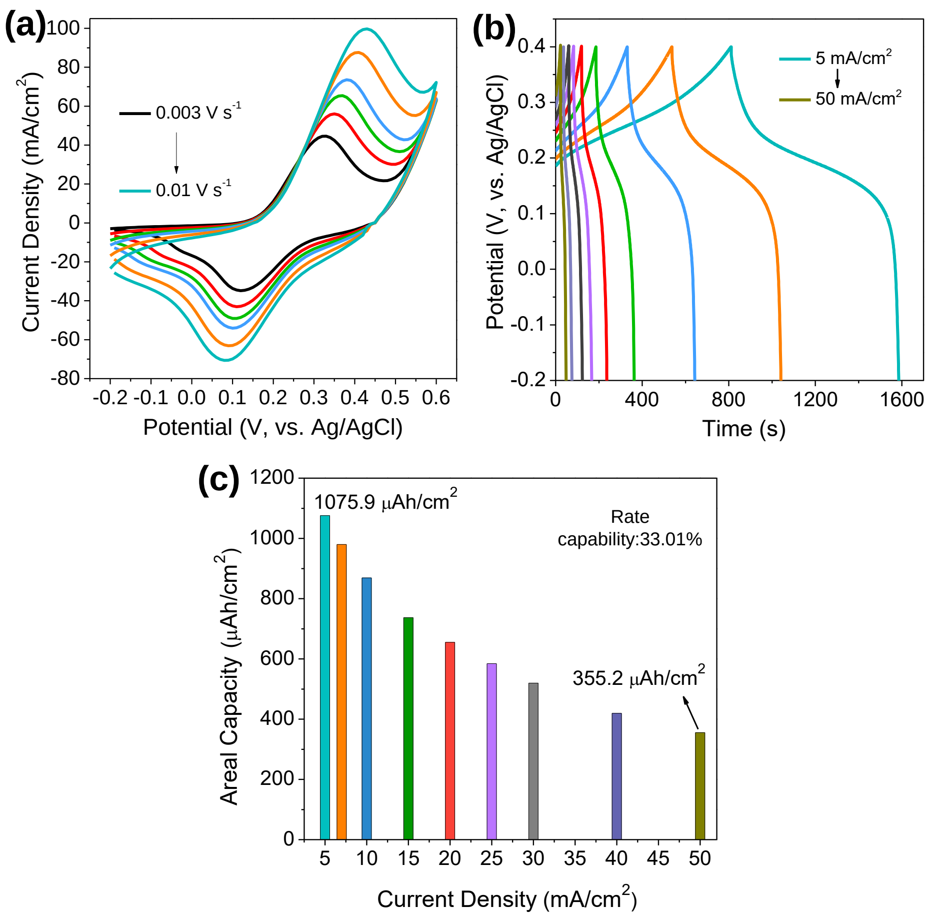

Fig. S5 Electrochemical properties of the MOF-based NCM-10 evaluated in 1 M KOH electrolyte. (**a**) CV curves evaluated at different scan rates of 0.003-0.01 V s^{-1}, (**b**) GCD curves measured at different current densities of 5-10 mA cm^{-2} and (**c**) caluclated capacity values with respect to the current densities of the MOF-based NCM-10

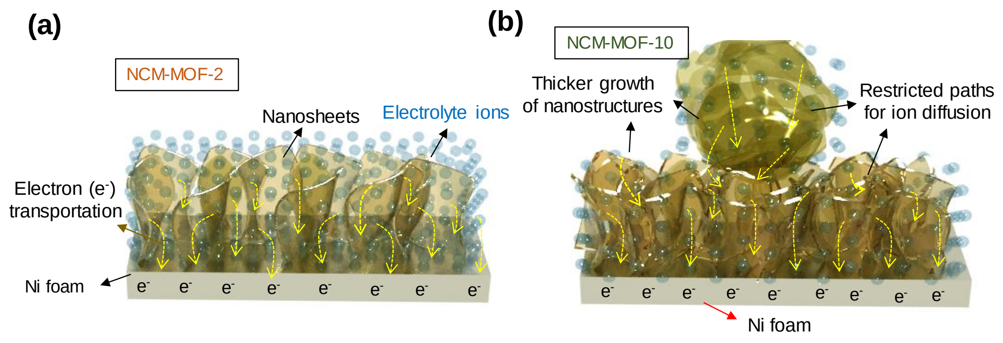

Fig. S6 Schematic prediction of electrochemical merits/dissontvages of (**a**) NCM-based MOF-2 and (**b**) NCM-based MOF-10 electrodes during the energy storage process

Table S1 Comparative energy storage performance of our dual layered NCM-based MOF with earlier reported metal oxide/sulfide and MOF-based materials in three-electrode system

**Electroactive material**	**Morphology**	**Electrolyte**	**Test condition ($mA\ cm^{-2}$)**	**Area (cm^2)**	**Areal capacity ($\mu Ah\ cm^{-2}$)**	**Refs.**
MOF-74 derived $Ni_xCo_{3-x}O_4$	Nanoparticles	6 M KOH	6	1	325.83	[S1]
Co-MOF	nanosheets	5 M KOH	4.5	1	1121.75	[S2]
Co-MOF derived Co_9S_8@S,N-doped carbon materials	Cuboid structure	6 M KOH	2.4	1	143	[S3]
Core–shell Ni-MOF-74@Co-MOF-74 derived Ni_3S_4@Co_9S_8	Shell-in-shell tubes	2 M NaOH	2.4	1	367.47	[S4]
Ni-MOF	Accordion-like nanosheets	3 M KOH	3.5	1	638.12	[S5]
Ni-MOF/CNTs	Nanosheets	6 M NaOH	2	1	784.44	[S6]
Ni-MOF	Sheet-like structure	6 M KOH	2.5	1	547.84	[S7]
MOF-derived NiO	Porous nanoparticle	6 M KOH	5	1	201.25	[S8]
MOF-derived NiO/ZnO	Hierarchical hollow spheres	3 M KOH	5.2	1	276.11	[S9]
Zn/Co-ZIF derived Zn-Co-S	Rhombic dodecahedral cages	6 M NaOH	1	1	175.83	[S10]
Ni-based MOF	Nanorods	2 M KOH	1.9	1	131.1	[S11]
Bimetallic NiCo-MOF-74	Spherical particles	6 M KOH	3	1	238.33	[S12]
Ni-MOF derived NiO	Hexagonal flakes	3 M KOH	2	2	74.09	[S13]

MOF derived Ni–Co–S	Hierarchical nanosheets	1 M KOH	0.45	1	123.10	[S14]
MOF-derived hollow porous $Ni_xP_yO_z$	Microrods	2 M KOH	1.5	1	305.06	[S15]
NCM-based MOF	Dual layered nanosheets/ nanoflowers	1 M KOH	5	1	1311.4	This work

Table S2 Comparative areal energy and power density values of recently reported hybrid devices with our dual layered NCM-based MOF//AC supercapattery

**Positive electrode material**	**Negative electrode material**	**Potential window**	**Areal E_d ($mWh\ cm^{-2}$)**	**Areal P_d ($mW\ cm^{-2}$)**	**Refs.**
Accordion-like Ni-MOF superstructure	Activated carbon	1.2 V	0.046	0.6	[S5]
Ni-MOF/CNTs	rGO/g-C_3N_4	1.6 V	0.1648	1.8	[S6]
Onion-like nanoporous $CuCo_2O_4$ hollow spheres	Activated carbon	1.5 V	0.2437	3.75	[S16]
MOF derived Ni–Co–S	Activated carbon	1.7 V	0.0955	3.27	[S14]
MOF derived MnO_x–MHCF nanocubes	Activated carbon	1.3 V	0.0410	0.32	[S17]
Hierarchical porous Ni-MOF	Activated carbon	1.4 V	0.0971	1.43	[S18]
Ni-based MOF	Activated carbon	1.2 V	0.2	1.8	[S19]
MOF derived Nanoporous Co_3O_4	Nanoporous Carbon	1.6 V	0.0718	3.2	[S20]
ZnO QDs/carbon/CNTs	N-doped carbon/CNTs	1.7 V	0.1030	3.69	[S21]
Ni-Co MOF	Activated carbon		0.64	2.57	[S22]
Co-Mn MOFs	Activated carbon	1.6	0.12	4.68	[S23]
Dual layered NCM-based MOFs	Activated carbon	1.55 V	1.21	5.3	This work

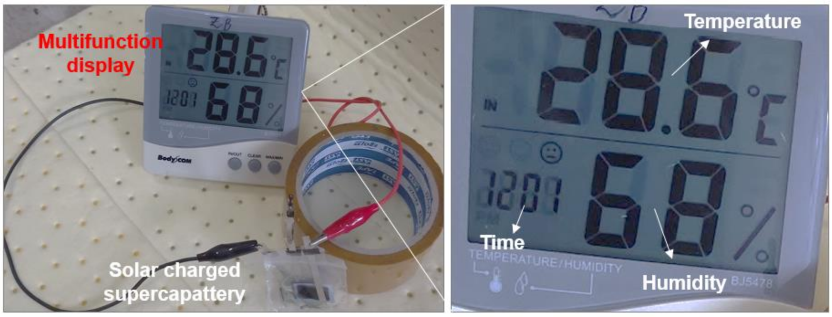

Fig. S7 Powering multifunction electronic display by solar charge solitary supercapattery

Supplementary References

[S1] S. Chen, M. Xue, Y. Li, Y. Pan, L. Zhu, S. Qiu, Rational design and synthesis of nixco3−xo4 nanoparticles derived from multivariate mof-74 for supercapacitors. J. Mater. Chem. A **3**(40), 20145-20152 (2015). https://doi.org/10.1039/C5TA02557E

[S2] J. Yang, Z. Ma, W. Gao, M. Wei, Layered structural Co-based MOF with conductive network frames as a new supercapacitor electrode. Chem. Eur. J. **23**(3), 631-636 (2017). https://doi.org/10.1002/chem.201604071

[S3] S. Liu, M. Tong, G. Liu, X. Zhang, Z. Wang et al., N-containing Co-MOF derived Co_9S_8@S,N-doped carbon materials as efficient oxygen electrocatalysts and supercapacitor electrode materials. Inorg. Chem. Front. **4**(3), 491-498 (2017). https://doi.org/10.1039/C6QI00403B

[S4] H. Li, F. Yue, H. Xie, C. Yang, Y. Zhang, L. Zhang, J. Wang, Hollow shell-in-shell Ni_3S_4@Co_9S_8 tubes derived from core–shell Ni-MOF-74@Co-MOF-74 as efficient faradaic electrodes. CrystEngComm **20**(7), 889-895 (2018). https://doi.org/10.1039/C7CE01873H

[S5] Y. Yan, P. Gu, S. Zheng, M. Zheng, H. Pang, H. Xue, Facile synthesis of an accordion-like Ni-MOF superstructure for high-performance flexible supercapacitors. J. Mater. Chem. A **4**(48), 19078-19085 (2016). https://doi.org/10.1039/C6TA08331E

[S6] P. Wen, P. Gong, J. Sun, J. Wang, S. Yang, Design and synthesis of Ni-MOF/CNT composites and RGO/carbon nitride composites for an asymmetric supercapacitor with high energy and power density. J. Mater. Chem. A **3**(26), 13874-13883 (2015). https://doi.org/10.1039/C5TA02461G

[S7] J. Yang, P. Xiong, C. Zheng, H. Qiu, M. Wei, Metal–organic frameworks: A new promising class of materials for a high performance supercapacitor electrode. J. Mater. Chem. A **2**(39), 16640-16644 (2014). https://doi.org/10.1039/C4TA04140B

[S8] Y. Han, S. Zhang, N. Shen, D. Li, X. Li, MOF-derived porous NiO nanoparticle architecture for high performance supercapacitors. Mater. Lett. **188**, 1-4 (2017). https://doi.org/10.1016/j.matlet.2016.09.051

[S9] G.-C. Li, P.-F. Liu, R. Liu, M. Liu, K. Tao et al., MOF-derived hierarchical double-shelled NiO/ZnO hollow spheres for high-performance supercapacitors. Dalton Trans. **45**(34), 13311-13316 (2016). https://doi.org/10.1039/C6DT01791F

[S10] P. Zhang, B.Y. Guan, L. Yu, X.W. Lou, Formation of double-shelled zinc–cobalt sulfide dodecahedral cages from bimetallic zeolitic imidazolate frameworks for hybrid supercapacitors. Angew. Chem. Int. Ed. **56**(25), 7141-7145 (2017). https://doi.org/10.1002/anie.201702649

[S11] C. Qu, Y. Jiao, B. Zhao, D. Chen, R. Zou, K.S. Walton, M. Liu, Nickel-based pillared MOFs for high-performance supercapacitors: Design, synthesis and stability study. Nano Energy **26**, 66-73 (2016). https://doi.org/10.1016/j.nanoen.2016.04.003

[S12] C. Young, J. Kim, Y.V. Kaneti, Y. Yamauchi, One-step synthetic strategy of hybrid materials from bimetallic metal–organic frameworks for supercapacitor applications. ACS Appl. Energy Mater. **1**(5), 2007-2015 (2018). https://doi.org/10.1021/acsaem.8b00103

[S13] S. Gao, Y. Sui, F. Wei, J. Qi, Q. Meng, Y. He, Facile synthesis of nickel metal–organic framework derived hexagonal flaky nio for supercapacitors. J. Mater. Sci. - Mater. Electron. **29**(3), 2477-2483 (2018). https://doi.org/10.1007/s10854-017-8169-7

[S14] K. Tao, X. Han, Q. Ma, L. Han, A metal–organic framework derived hierarchical nickel–cobalt sulfide nanosheet array on ni foam with enhanced electrochemical performance for supercapacitors. Dalton Trans. **47**(10), 3496-3502 (2018). https://doi.org/10.1039/C7DT04942K

[S15] R. Bendi, V. Kumar, V. Bhavanasi, K. Parida, P.S. Lee, Metal organic framework-derived metal phosphates as electrode materials for supercapacitors. Adv. Energy Mater. **6**(3), 1501833 (2016). https://doi.org/10.1002/aenm.201501833

[S16] A.A. Ensafi, S.E. Moosavifard, B. Rezaei, S.K. Kaverlavani, Engineering onion-like nanoporous $CuCo_2O_4$ hollow spheres derived from bimetal–organic frameworks for high-performance asymmetric supercapacitors. J. Mater. Chem. A **6**(22), 10497-10506 (2018). https://doi.org/10.1039/C8TA02819B

[S17] Y.-Z. Zhang, T. Cheng, Y. Wang, W.-Y. Lai, H. Pang, W. Huang, A simple approach to boost capacitance: Flexible supercapacitors based on manganese oxides@MOFs via chemically induced in situ self-transformation. Adv. Mater. **28**(26), 5242-5248 (2016). https://doi.org/10.1002/adma.201600319

[S18] P. Du, Y. Dong, C. Liu, W. Wei, D. Liu, P. Liu, Fabrication of hierarchical porous nickel based metal-organic framework (Ni-MOF) constructed with nanosheets as novel pseudo-capacitive material for asymmetric supercapacitor. J. Colloid Interface Sci. **518**, 57-68 (2018). https://doi.org/10.1016/j.jcis.2018.02.010

[S19] L. Kang, S.-X. Sun, L.-B. Kong, J.-W. Lang, Y.-C. Luo, Investigating metal-organic framework as a new pseudo-capacitive material for supercapacitors. Chin. Chem. Lett. **25**(6), 957-961 (2014). https://doi.org/10.1016/j.cclet.2014.05.032

[S20] R.R. Salunkhe, J. Tang, Y. Kamachi, T. Nakato, J.H. Kim, Y. Yamauchi, Asymmetric supercapacitors using 3d nanoporous carbon and cobalt oxide electrodes synthesized from a single metal–organic framework. ACS Nano **9**(6), 6288-6296 (2015). https://doi.org/10.1021/acsnano.5b01790

[S21] Y. Zhang, B. Lin, J. Wang, J. Tian, Y. Sun, X. Zhang, H. Yang, All-solid-state asymmetric supercapacitors based on ZnO quantum dots/carbon/CNT and porous n-doped carbon/CNT electrodes derived from a single ZIF-8/CNT template. J. Mater. Chem. A **4**(26), 10282-10293 (2016). https://doi.org/10.1039/C6TA03633C

[S22] Y. Wang, Y. Liu, H. Wang, W. Liu, Y. Li et al., Ultrathin NiCo-MOF nanosheets for high-performance supercapacitor electrodes. ACS Appl. Energy Mater. **2**(3), 2063-2071 (2019). https://doi.org/10.1021/acsaem.8b02128

[S23] S.H. Kazemi, B. Hosseinzadeh, H. Kazemi, M.A. Kiani, S. Hajati, Facile synthesis of mixed metal–organic frameworks: Electrode materials for supercapacitors with excellent areal capacitance and operational stability. ACS Appl. Mater. Interfaces **10**(27), 23063-23073 (2018). https://doi.org/10.1021/acsami.8b04502
